# Scanning Probe Nano‐Infrared Imaging and Spectroscopy of Biochemical and Natural Materials

**DOI:** 10.1002/smsc.202400297

**Published:** 2024-09-26

**Authors:** Jialiang Shen, Byung‐Il Noh, Pengyu Chen, Siyuan Dai

**Affiliations:** ^1^ Materials Research and Education Center Department of Mechanical Engineering Auburn University Auburn Alabama 36849 USA

**Keywords:** biomaterials, infrared spectroscopy, natural specimens, near‐field optics, scanning probe

## Abstract

The mid‐infrared with a characteristic wavelength of 3–20 μm is important for a wealth of technologies. In particular, mid‐infrared spectroscopy can reveal material composition and structure information by fingerprinting chemical bonds’ infrared resonances. Despite these merits, state‐of‐the‐art mid‐infrared techniques are spatially limited above tens of micrometers due to the fundamental diffraction law. Herein, recent progress in the scanning probe nanoscale infrared characterization of biochemical materials and natural specimens beyond this spatial limitation is reviewed. By leveraging the strong tip–sample local interactions, scanning probe nano‐infrared methods probe nanoscale optical and mechanical responses to disclose material composition, heterogeneity, orientation, fine structure, and phase transitions at unprecedented length scales. These advances, therefore, revolutionize the understanding of a broad range of biochemical and natural materials and offer new material manipulation and engineering opportunities close to the ultimate length scales of fundamental physical, chemical, and biological processes.

## Graphical abstract

By leveraging the strong tip–sample local interactions, scanning probe nano‐infrared methods probe nanoscale material optical and mechanical responses. These unprecedented information revolutionize the understanding of a broad range of biochemical and natural materials and offer new material manipulation and engineering opportunities close to the ultimate length scales of fundamental physical, chemical, and biological processes.

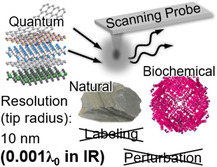

## 1 Introduction

The mid‐infrared (mid‐IR)^[^ ^1^, ^2^, ^3^, ^4^, ^5^, ^6^, ^7^ ^]^ is an important wavelength range (3–20 μm and frequency 500–3333 cm^−1^) for a wealth of technologies, including biochemical sensing,^[^ ^1^, ^2^, ^8^, ^9^ ^]^ gas monitoring,^[^ ^3^, ^4^ ^]^ thermal illumination,^[^ ^10^, ^11^ ^]^ energy control,^[^ ^12^ ^]^ security and defense,^[^ ^13^ ^]^ and many others.^[^ ^5^, ^6^, ^14^ ^]^ In particular, mid‐IR spectroscopy reveals composition and structural information based on the resonances of specific wavelengths of mid‐IR light with materials’ chemical bonds—the mid‐IR “fingerprinting.”^[^ ^15^ ^]^ Compared with the UV (190–400 nm) and visible (400–800 nm) light, the mid‐IR light has a longer wavelength and lower frequency, hence less energy. Consequently, the mid‐IR light cannot excite electrons to higher energy levels like the UV and visible counterparts. Instead, mid‐IR light excites vibrations and rotations of covalent bonds in molecules that are classified by chemical groups.^[^ ^16^ ^]^ Each chemical group resonates with mid‐IR light at specific wavelengths characteristic of that chemical moiety.^[^ ^17^ ^]^ Therefore, the versatility of mid‐IR spectroscopy allows for identifying functional groups in materials ranging from biomedical devices^[^ ^18^, ^19^ ^]^ to natural samples,^[^ ^20^, ^21^ ^]^ food, and many others.^[^ ^15^, ^22^ ^]^

Despite various valuable applications in chemical identification, material diagnosis, and monitoring, mid‐IR spectroscopy faces a fundamental limitation in its spatial resolution of ≈3–10 μm. This limitation arises from the diffraction law due to the wave‐like nature of light: the diffraction of light waves limits the smallest distance between two resolvable points to be about the wavelength of light.^[^ ^23^ ^]^ In the mid‐IR, this limitation is ≈3–10 μm. As a result, material composition and structural information from current mid‐IR fingerprinting spectroscopy are global responses averaged over considerably large areas. As the ultimate length scales of fundamental physical, chemical, and biological processes are at the sub‐micro or nanometers, fruitful local composition and structural information inherent in the nonuniform nature of materials remain inaccessible using conventional mid‐IR spectroscopy.

Therefore, exploring materials’ nanoscale composition and structure requires special optical methods at subdiffractional length scales. One recent approach is integrating the scanning probe into mid‐IR spectroscopy for super‐resolution nano‐IR imaging and spectroscopy. This method only probes light at the tip apex of the nanoscale scanning probe, thus providing sample optical responses (reflection, absorption, etc.) with a spatial resolution of ≈10 nm, > 100 times better than conventional mid‐IR spectroscopy. In addition, the scanning probe nano‐IR is label‐ and perturbation‐free, and its nano‐optical resolution is independent of the free space light wavelength. With all these advances, the scanning probe nano‐IR imaging and spectroscopy have been exploited to disclose a variety of important nano‐optical phenomena and reveal nanoscale composition and structural information on diverse samples, including quantum,^[^ ^24^, ^25^, ^26^, ^27^, ^28^, ^29^, ^30^, ^31^, ^32^, ^33^, ^34^, ^35^, ^36^, ^37^, ^38^, ^39^, ^40^, ^41^, ^42^, ^43^, ^44^, ^45^, ^46^, ^47^, ^48^, ^49^, ^50^ ^]^ catalytic,^[^ ^51^, ^52^, ^53^ ^]^ energy‐storage,^[^ ^54^, ^55^, ^56^ ^]^ natural,^[^ ^57^, ^58^, ^59^, ^60^, ^61^, ^62^, ^63^, ^64^, ^65^ ^]^ and bio^[^ ^66^, ^67^, ^68^, ^69^, ^70^, ^71^, ^72^, ^73^, ^74^, ^75^, ^76^, ^77^, ^78^ ^]^ materials.

Here, we review recent progress in scanning probe nano‐IR imaging and spectroscopy studies of biomaterials and natural specimens. In Section 1, we overview the importance and spatial limitation of current mid‐IR spectroscopy and the potential of scanning probe nano‐IR to overcome this limitation. Section 2 describes the state‐of‐the‐art scanning probe nano‐IR methods by introducing their operational mechanisms and performance metrics. In Section 3, we highlight representative studies using scanning probe nano‐IR on various biomaterials and natural specimens. Finally, a summary and a brief perspective on the future of scanning probe nano‐IR are provided in Section 4.

## 2 Overview of Scanning Probe Nano‐IR Techniques

**Figure 1:**
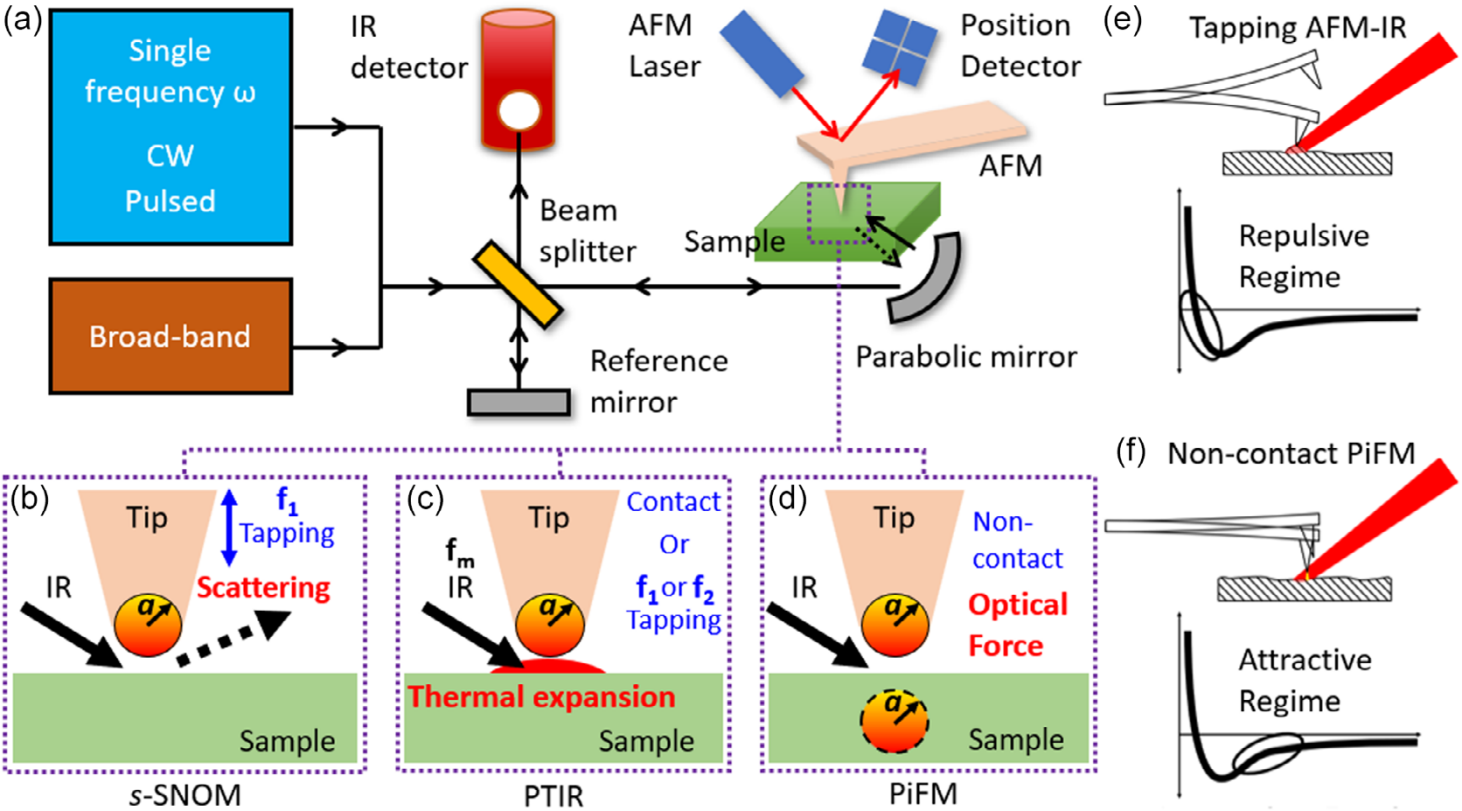
Schematics of scanning probe nano‐IR techniques. a) Experimental setup. b–d) Detection mechanisms include: b) collecting optical signals through light scattering, known as s‐SNOM; monitoring mechanical deflections c) due to thermal expansion referred to as PTIR (using AFM contact mode) or tapping AFM‐IR (using AFM tapping mode at *f*_1_ or *f*_2_, IR laser modulates at *f*_m_ = *f*_2_ − *f*_1_), and d) based on optical force referred to as PiFM. Optical detection requires IR detector, beam splitter, and reference mirror, whereas mechanical detection relies on the deflection signal of the AFM cantilever. e) Tapping AFM‐IR operates within the repulsive region. f) PiFM works in the attractive regime, utilizing the noncontact AFM mode. e–f) Reproduced with permission.^[^ ^105^ ^]^ Copyright 2024, Molecular Vista.

In the scanning probe nano‐IR experiments, a sharp atomic force microscopy (AFM) tip is illuminated by the free‐space light. As an antenna, the illuminated tip generates a strong electromagnetic near‐field confined at the tip apex. This strong near‐field interacts vigorously with the underneath scanned sample whose nanoscale IR responses are detected optically or mechanically (**Figure** **1**a). The optical detection, typically referred to as scattering‐type scanning near‐field optical microscopy (s‐SNOM), utilizes an optical detector in the far field to record tip‐scattered IR light related to samples’ local reflection and absorption. In contrast, the mechanical detection, including photothermal‐induced resonance (PTIR) spectroscopy,^[^ ^79^, ^80^, ^81^, ^82^, ^83^, ^84^ ^]^ tapping mode PTIR or tapping AFM‐IR, photoinduced force microscopy (PiFM),^[^ ^85^, ^86^, ^87^, ^88^ ^]^ and peak force infrared (PFIR) microscopy,^[^ ^89^, ^90^, ^91^, ^92^, ^93^ ^]^ records the cantilever deflection to reveal samples’ local thermal expansion, photoinduced forces, and other mechanical responses caused by the IR responses.

The s‐SNOM utilizes a tapping AFM (Figure 1b) to offer soft material compatibility and lock‐in amplifications for the local optical signal at the tip apex. The latter can be isolated from the sample's global response by demodulating at high harmonics of the tapping frequency. Moreover, the s‐SNOM implements an oscillating reference mirror—the pseudo‐heterodyne detection^[^ ^94^ ^]^—to further refine the near‐field response and, more importantly, separate the amplitude and phase of the complex optical signal, which relates to the sample's reflection and absorption,^[^ ^95^, ^96^, ^97^, ^98^, ^99^ ^]^ respectively. In the experiment, the s‐SNOM can be illuminated by a monochromatic laser to perform IR nanoimaging. In addition, a broadband light source can be used for nanoscale Fourier transform IR spectroscopy (nano‐FTIR)^[^ ^77^, ^100^ ^]^ by scanning the reference mirror.

Mechanical‐detection scanning probe nano‐IR does not require an IR optical detector but monitors the cantilever deflection for samples’ nanoscale IR responses. The PTIR (Figure 1c) measures the sample's local thermal expansion proportional to the IR absorption. Early PTIR exploits a contact mode AFM illuminated by a low‐repetition (≈1 kHz) pulsed laser.^[^ ^88^, ^101^, ^102^ ^]^ The low‐repetition pulse creates periodically IR‐induced responses between sample and tip and localizes the IR absorption of the sample, thereby leading to a high spatial resolution. As a result, the AFM must be operated in contact mode to adequately detect the weak IR absorption through thermal expansion. While successful,^[^ ^88^, ^101^, ^102^, ^103^ ^]^ contact‐mode PTIR may cause specimen damage, especially for soft, mobile, or delicate samples, due to the AFM tip's lateral frictional force during the scan. In addition, contact mode PTIR may not yield genuine IR responses because the deflection signal can originate from not only thermal expansion but also material surface variations. To overcome these difficulties, tapping mode PTIR (tapping AFM‐IR) was recently developed. In order to compensate for the signal loss in contact‐to‐tapping mode conversion, the illumination laser is modulated at the frequency difference between the cantilever's first and second resonances (*f*_m_ = *f*_2_ − *f*_1_) to enable the sideband detection through the frequency mixing, thereby amplifying the excitation and detection of the IR signal by the resonance quality factor. In tapping AFM‐IR, the AFM is still operated at *f*_1_ or *f*_2_, so nano‐IR signal and sample topography can be extracted at different resonances to avoid artifacts in both parameters.

It is worth mentioning that based on PTIR and tapping AFM‐IR, two useful scanning probe nano‐IR techniques—PiFM and PFIR^[^ ^93^ ^]^—have emerged recently. PiFM^[^ ^88^, ^104^ ^]^ operates the AFM in the noncontact mode and monitors the photoinduced force, instead of the thermal expansion (e.g., PTIR and tapping AFM‐IR), to detect materials’ nanoscale optical responses. The photoinduced force, originating from the attractive dipole–dipole interaction (Figure 1d) between the AFM tip and the sample, highly depends on the tip–sample distance. Therefore, the lock‐in detection of the cantilever deflection at the tip's oscillation resonance can effectively isolate the photoinduced force. Instead of the repulsive tip–sample interaction^[^ ^105^ ^]^ in Tapping AFM‐IR (Figure 1e), PiFM possesses an attractive photoinduced force (Figure 1f) in the noncontact mode, thereby offering better compatibility with materials of low thermal expansion and soft, mobile, and delicate samples. In addition, PFIR taps the cantilever on samples at a slow tip‐force frequency to measure samples’ mechanical responses, including the elasticity and adhesion, in addition to the IR absorption through the thermal expansion.

## 3 Nanoscale Chemical Identification

### 3.1 Nanostructure of Polymer Blends

**Figure 2:**
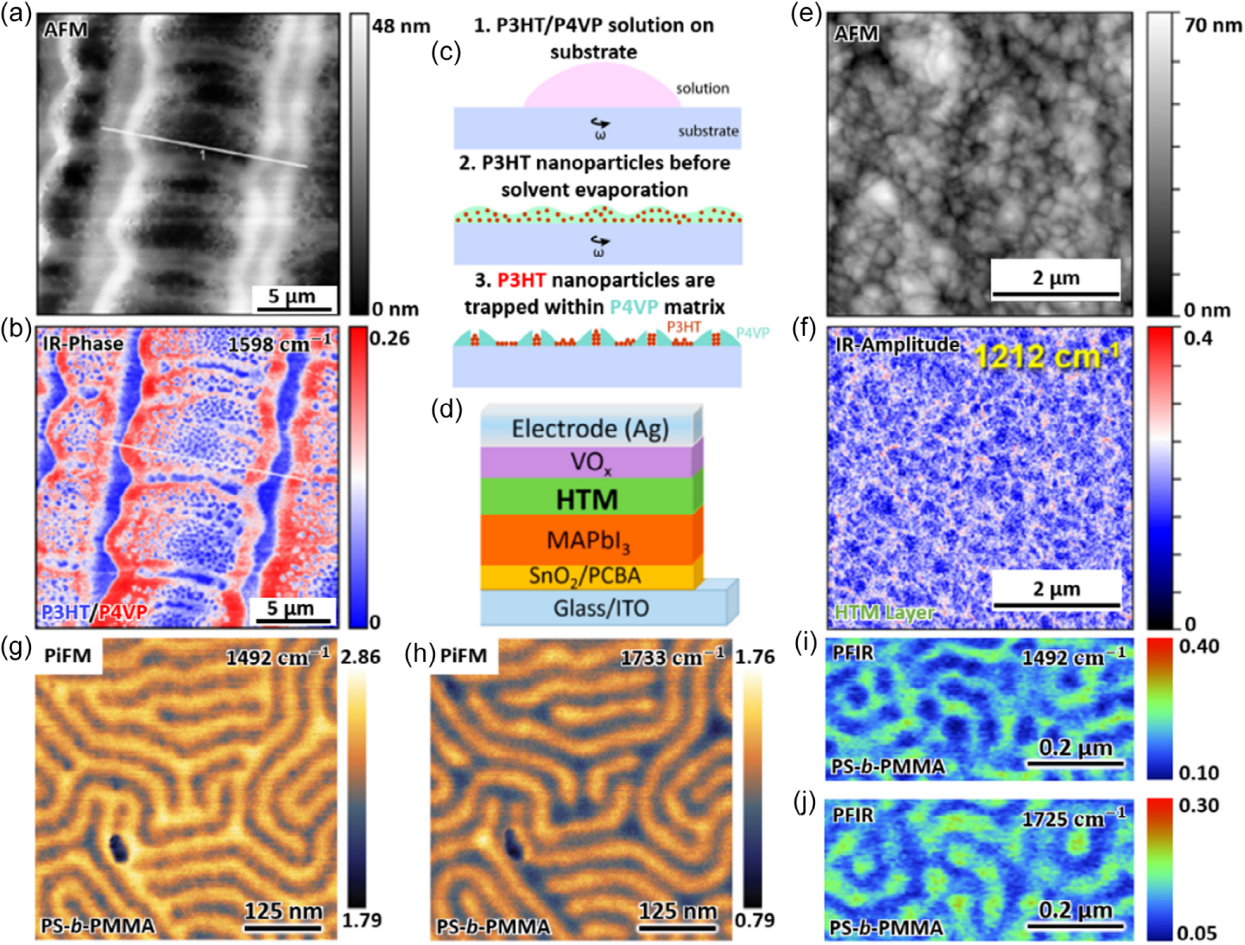
IR nanoimaging characterization of the polymer heterogeneity. a) AFM and b) s‐SNOM IR phase images of P3HT/P4VP polymer blends at 1598 cm^−1^ (the P4VP ring vibrational mode). Regions with low phase contrast (“blue”) correspond to P3HT, while areas with high phase contrast (“red”) correspond to P4VP. c) A possible sequence of solvent evaporation processes during the self‐assembly of polymer film formation. Reproduced with permission.^[^ ^106^ ^]^ Copyright 2022, American Chemical Society. d) Device architecture of the organic solar cell. e) AFM and f) s‐SNOM IR amplitude image of the HTM/MAPbI_3_/Glass sample at 1212 cm^−1^ which is the characteristic IR frequency of HTM layer. Variations in the IR amplitude indicate the inhomogeneity of HTM layer. Reproduced with permission.^[^ ^108^ ^]^ Copyright 2022, American Chemical Society. g,h) PiFM images at 1492 and 1733 cm^−1^, and i,j) PFIR images of PS‐b‐PMMA copolymer at 1492 and 1725 cm^−1^, which correspond to resonant frequencies of PS and PMMA, respectively. Reproduced with permission.^[^ ^88^, ^93^ ^]^ Copyright 2016, AAAS; Copyright 2017, AAAS.

Nanoscale chemical information discloses material fine structure and offers insights into their chemical and biological functionalities.^[^ ^82^, ^83^, ^88^ ^]^ As a representative example (**Figure** **2**a–c), s‐SNOM phase imaging was utilized to study self‐assembled polymer blends comprising poly(3‐hexylthiophene‐2,5‐diyl) (P3HT) and poly(4‐vinylpyridine) (P4VP) and reveal the nanoscale heterogeneity of the polymers. This heterogeneity is particularly evident at the P4VP ring vibrational mode at 1598 cm^−1^ (Figure 2b). These results offer important insights into the solvent evaporation processes during the polymer self‐assembly, where P3HT precipitates into nanoparticles and are trapped inside the P4VP strips (Figure 2c).^[^ ^106^ ^]^ In addition, s‐SNOM amplitude imaging has also been utilized to study the nanoscale heterogeneity of conjugated polymers—the hole‐transport materials (HTM) layers in organic and perovskite solar cells (Figure 2d). The nano‐IR image (Figure 2f) reveals nanoscale material composites^[^ ^107^ ^]^ and voids or pinholes in the HTM layer.^[^ ^108^ ^]^ Notably, the material heterogeneity provides additional scattering to the charge carriers, thereby affecting the photovoltaic performance. Understanding the nanoscale heterogeneity through the scanning probe nano‐IR data will offer valuable guidance in designing and optimizing organic solar cells. In addition to s‐SNOM, PiFM^[^ ^88^ ^]^ (Figure 2g,h) and PFIR^[^ ^93^ ^]^ (Figure 2i,j) can also reveal the nanoscale polymer heterogeneity: Polystyrene (PS) and polymethyl methacrylate (PMMA) can be identified at the nanoscale at their resonance of 1492 and 1725 cm^−1^, respectively.

### 3.2 Orientation and Crystallinity of Polymers

**Figure 3:**
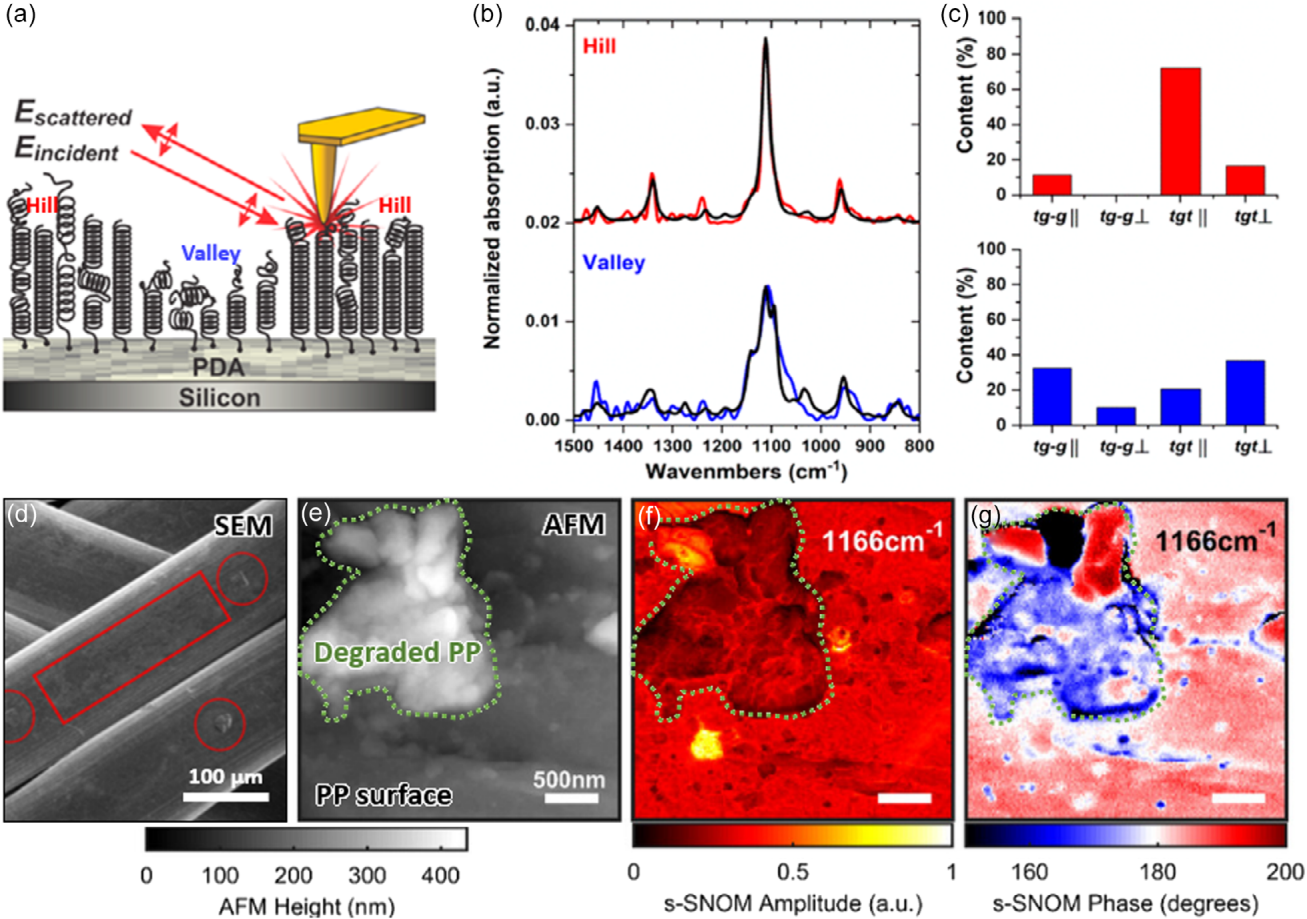
Characterizations of polymer‐chain orientation and polymer degradation. a) Schematic of nano‐IR technique and conformations of polymer brush coatings. b) Absorption spectra for hill (red curve) and valley (blue curve) regions, with the black curves representing simulated spectra for the corresponding regions. c) Calculated content of orientations (|| and ⊥ refer parallel and perpendicular to the AFM tip, respectively) for two conformers (tg‐g and tgt) of chains in hill and valley, respectively. Reproduced with permission.^[^ ^111^ ^]^ Copyright 2020, American Chemical Society. d) SEM image of PP after degradation, with areas of surface degradation highlighted by a rectangular box and circles. e) AFM, f) s‐SNOM amplitude, and g) phase images of degraded PP samples at 1166 cm^−1^. Dashed curve marked region in e–g) shows degraded PP particle, which exhibits higher topographic variation and varied phase contrast compared to the undegraded surrounding area. Reproduced with permission.^[^ ^112^ ^]^ Copyright 2023, Wiley‐VCH.

In addition to identifying compositions through characteristic vibration resonances, scanning probe nano‐IR can reveal the orientations of polymer‐chain vibrations. Typically, the scanning probe tip is illuminated by p‐polarized light, which preferentially excites vertical molecular vibrations. This characteristic is leveraged to examine the orientation of molecular vibrations.^[^ ^84^, ^109^, ^110^ ^]^ For instance, in **Figure** **3**a–c, the nanoscale distribution of chain orientations in polymer brush coatings was elucidated by nano‐FTIR. Figure 3a highlights the topographic variation within coated polymer film, revealing the alternating of low and high topographic regions, termed “valley” and “hill,” respectively. The nano‐FTIR results (Figure 3c) indicate that polymer chains in “hill” regions mainly aligned parallel to the tip, whereas those in the “valley”’ regions aligned perpendicularly.^[^ ^111^ ^]^

Furthermore, scanning probe nano‐IR is capable of characterizing the crystallinity of polymers, as demonstrated in Figure 3d–g. This technique was applied to study the degradation of polypropylene (PP) surgical mesh, a device that releases PP particles during surgeries to provoke cellular irritation and immune response. Degraded PP particles exhibit a loss of C—C bond, reflecting a decrease in PP crystallinity. This results in enhanced absorption from the side‐group r(CH_3_), thus creating a discernible phase contrast in the degraded areas, as shown in Figure 3g. The released PP particles (green dashed region) possess amorphous C—H bonds, thus exhibiting different (either higher or lower) IR phase contrast from the undegraded surroundings^[^ ^112^ ^]^ at the 1166 cm^−1^ resonance.

### 3.3 Dependence on Sample Thickness with Nanometer Sensitivity

**Figure 4:**
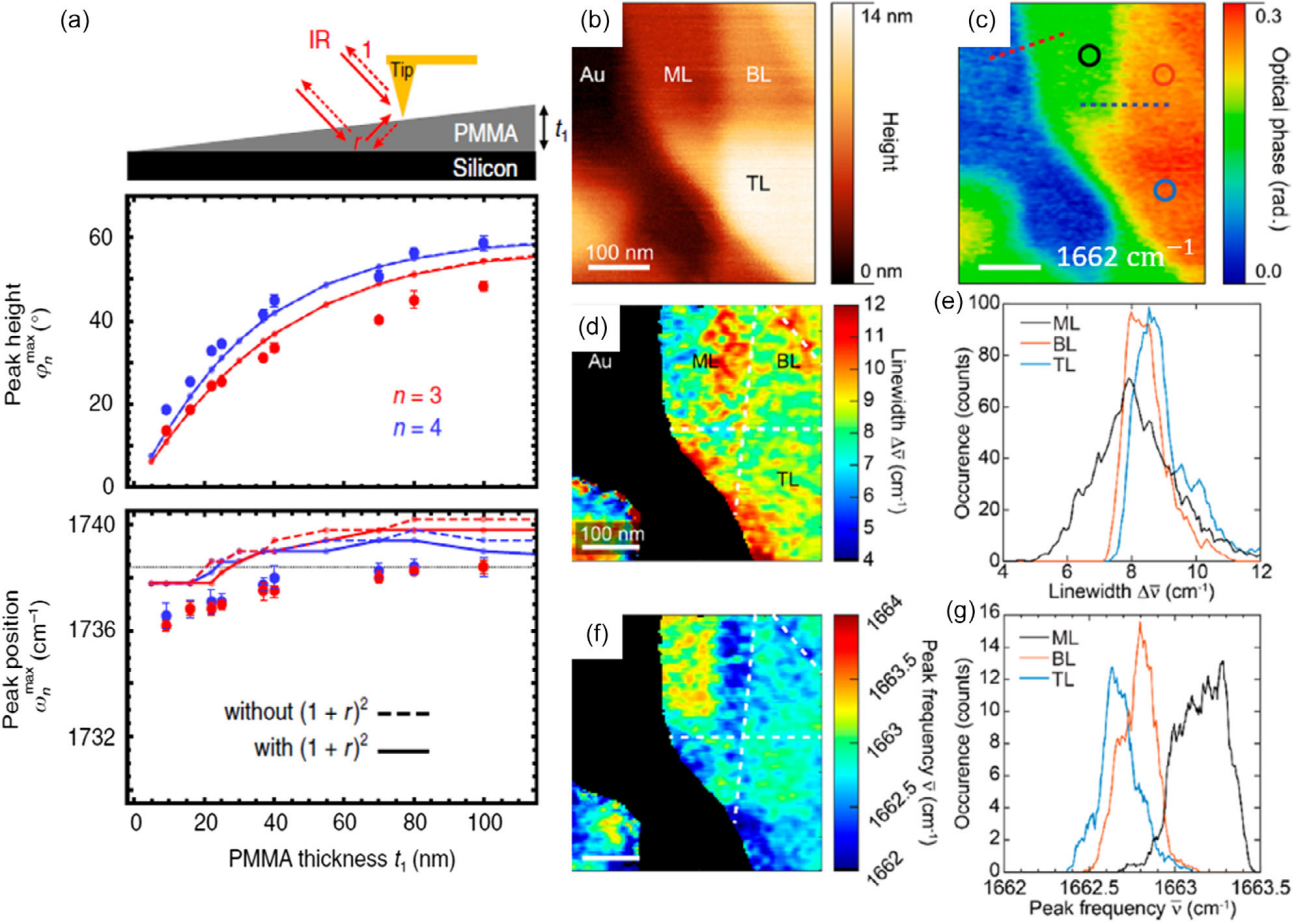
Thickness of polymer with nanometer sensitivity. a) Spectra peak heights and positions of nano‐FTIR spectra with the thickness of PMMA layers. Dots are experimental data and curves are calculated result, for 3 (red) and 4 (blue) orders of demodulation. Dashed and solid curves refer to the consideration without and with far‐field factor (1 + *r*)^2^, respectively. The horizontal dotted line is the peak position of absorption spectra for bulk PMMA samples. Reproduced with permission.^[^ ^113^ ^]^ Copyright 2020, Springer Nature. b,c) The AFM and s‐SNOM phase image at 1662 cm^−1^ on the peptoid sheets. d,f) The spectral linewidth and peak frequency of absorption spectra at each pixel on the spatial map. Collecting pixel information on regions for each number of layers, e,g) are the histogram of linewidth and peak frequency for each number of layers. Reproduced with permission.^[^ ^73^ ^]^ Copyright 2018, American Chemical Society.

Scanning probe nano‐IR imaging and spectroscopy rely on the local/surface interactions between the probe and the sample. Therefore, the nano‐IR imaging and spectra data are strongly affected by the sample thickness. A representative example in **Figure** **4**a reveals an increasing nano‐FTIR resonance peak intensity (middle) and a blueshifting frequency (bottom) in PMMA thin films at increasing thickness.^[^ ^113^ ^]^ This strong thickness dependence was also demonstrated in another example where the nano‐IR phase image exhibits layer number dependence on amino acids (Figure 4b–g). The amide I resonance in multilayer peptide nanosheets shows a narrower linewidth (Figure 4d,e) and redshift (Figure 4f,g) from a monolayer.^[^ ^73^ ^]^ These results suggest the promise of scanning probe nano‐IR in sensing the local thickness of complex nanomaterials and devices.

### 3.4 Nanoscale Compositions in Natural Specimens

**Figure 5:**
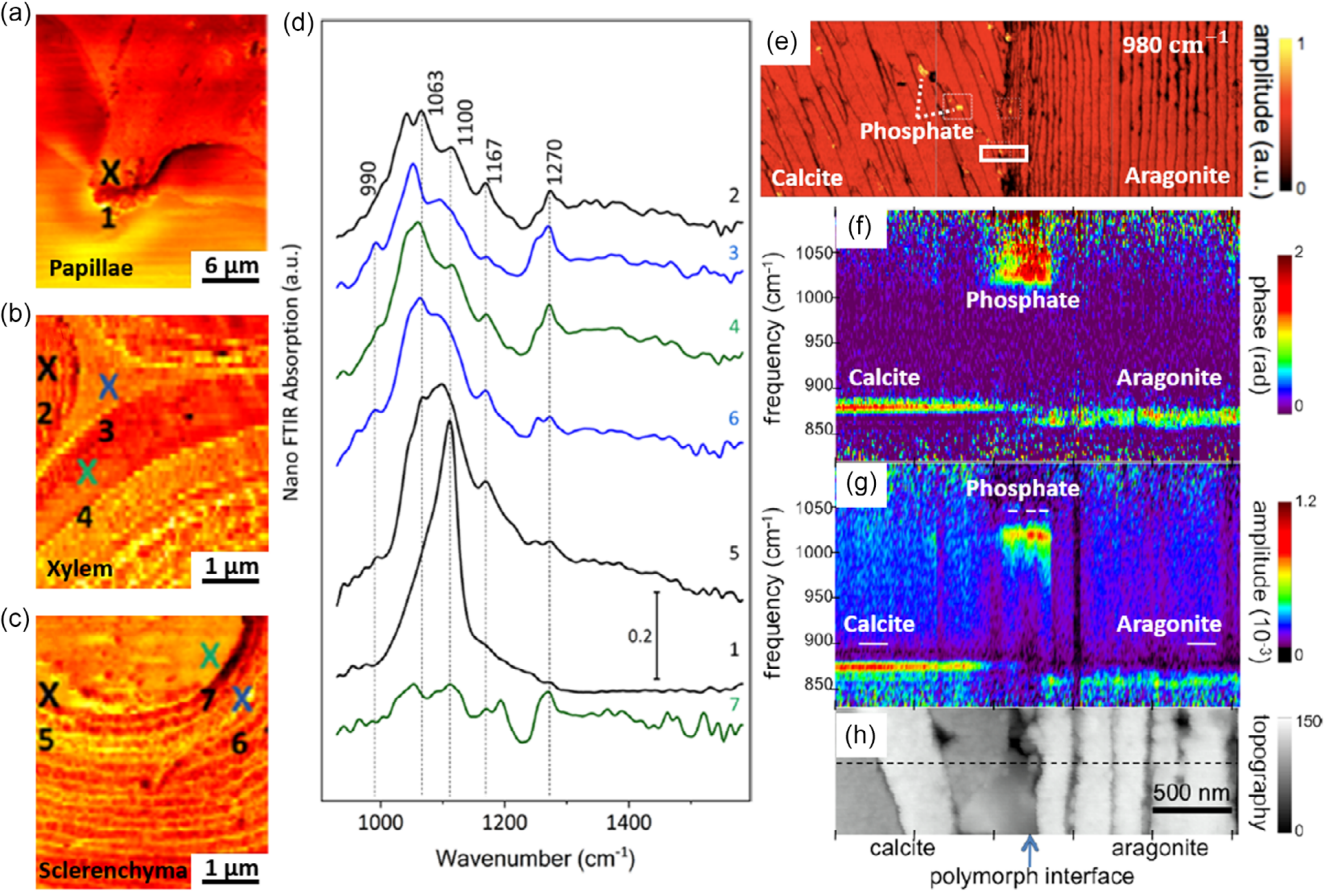
Characterizations of natural samples. a–c) Broadband near‐field images of a wheat awn at papilla, cell wall of xylem, and sclerenchyma, respectively. d) Nano‐FTIR absorption spectra were extracted at “X” marked positions in a–c). Reproduced with permission.^[^ ^58^ ^]^ Copyright 2020, American Chemical Society. e) s‐SNOM image of a polished *Mytilus edulis* shell at 980 cm^−1^. h) AFM topography measured at the region of solid white square in e) and phase and amplitude line profile of nano‐FTIR were extracted as f,g) which identified phonon resonance frequency of phosphate crystals at 1018 cm^−1^, calcite at 872 cm^−1^, and aragonite at 857 cm^−1^. Reproduced with permission.^[^ ^57^ ^]^ Copyright 2012, Beilstein‐Institut.

Scanning probe nano‐IR imaging and spectroscopy have been applied to characterize nanoscale compositions in natural specimens, e.g., shale rocks,^[^ ^65^ ^]^ meteorite,^[^ ^64^ ^]^ plants, and biominerals (**Figure** **5**), without complicated specimen preparations, including microsectioning, embedding, and sealing in conventional methods.^[^ ^58^, ^114^ ^]^ The local absorption through nano‐FTIR phase spectra has uncovered intricate nanoscale chemical compositions to elucidate the details of cell walls and phytolith in plants (Figure 5a–d).^[58^^]^ For instance, spot 1 in the papillae of wheat awn (Figure 5a) contains a significant amount of silica by exhibiting a spectral resonance at 1100 cm^−1^ in Figure 5d. The xylem tissue (spots 2, 3, and 4 in Figure 5b) is predominantly composed of starch or homogalacturonan by showing an evident C—C stretching bond at 1030–1060 cm^−1^. In addition, the sclerenchyma cell wall (spots 5 and 6) contains silica (1100 cm^−1^) and polysaccharides (1063 cm^−1^), whereas in the lumen of the sclerenchyma, no evident cellular features but epoxy resin's ether bond was detected at 1270 cm^−1^. Notably, spots 2, 3, and 4 (Figure 5b) are very close to each other (separations are ≈100 nm) but show different nano‐FTIR spectra, thus demonstrating the exceptional nanoscale spatial resolution of the scanning probe nano‐IR. In addition to the plants, scanning probe nano‐IR has been utilized to study biominerals (Figure 5e–h). The nanoscale distribution of crystalline phosphate (bright spots resonating at 1018 cm^−1^ in Figure 5e) was imaged in polished *Mytilus edulis* shells. Moreover, other mineral components, e.g., calcite at 872 cm^−1^ and aragonite at 857 cm^−1^,^[^ ^57^ ^]^ were also identified in the nanoscale.

### 3.5 Organics in Soil and Petroleum

**Figure 6:**
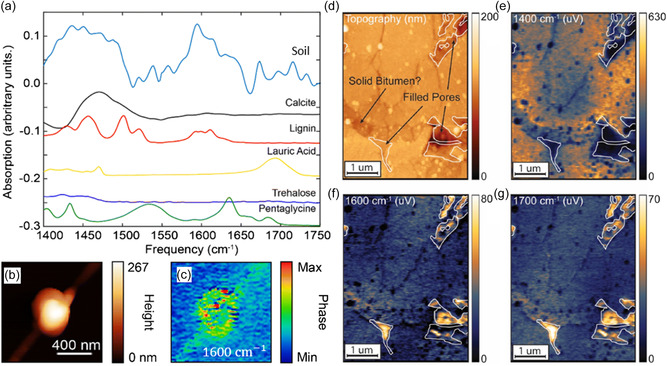
Characterization of organic matters in soil and petroleum samples. a) IR nanoabsorption spectra of a soil nanoparticle as blue curve, which is measured on the soil nanoparticle in panel b). Other curves are standard absorption spectra of possible pure components. b) AFM and c) phase image of s‐SNOM at 1600 cm^−1^ (aromatic stretch bond). Reproduced with permission.^[^ ^59^ ^]^ Copyright 2023, The Royal Society of Chemistry. d) Topography and near‐field amplitude images of Niobrara chalk sample with pores (marked by white curves) on calcite surface at e) 1400 cm^−1^ (C—O stretch resonance of calcite), f) 1600 cm^−1^, and g) 1700 cm^−1^ (carboxyl groups and carbon rings in oil migration), respectively. Reproduced with permission.^[^ ^115^ ^]^ Copyright 2021, Elsevier.

The scanning probe nano‐IR can characterize the chemical composition of soil organics to understand their nanoscale compositions. A representative example in **Figure** **6**a–c showcases the study of a soil nanoparticle by the scanning probe nano‐IR imaging and spectroscopy. The nano‐IR image (Figure 6c) reveals the soil nanoparticle's characteristic resonance at 1600 cm^−1^ by a strong absorption in the s‐SNOM phase. Detailed compositions of the soil nanoparticle can be disclosed by its nano‐FTIR spectrum (the light blue curve in Figure 6a, top). By fitting with the standard spectrum from calibration samples, calcite (black curve) and lignin (red curve) were found in the soil nanoparticle with the characteristic carbonate dominant stretch bond at ≈1450 cm^−1^ and aromatic stretch bond at ≈1600 cm^−1^, respectively.^[^ ^59^ ^]^

The scanning probe nano‐IR has also been utilized to study the oil–solid interactions in the petroleum industry. A representative Niobrara chalk sample exhibits pores on the surface (Figure 6d) with a different chemical nature from the undamaged calcite minerals, showing a lack of evident C—O stretch resonance at 1400 cm^−1^ (Figure 6e). The formation of the pores can be attributed to the oil migration because carboxyl groups and carbon rings were discovered inside the pores at the characteristic resonances of 1600 cm^−1^ (Figure 6f) and 1700 cm^−1^ (Figure 6g), respectively.^[^ ^115^ ^]^

### 3.6 Characterization of Material Phases at the Nanoscale

**Figure 7:**
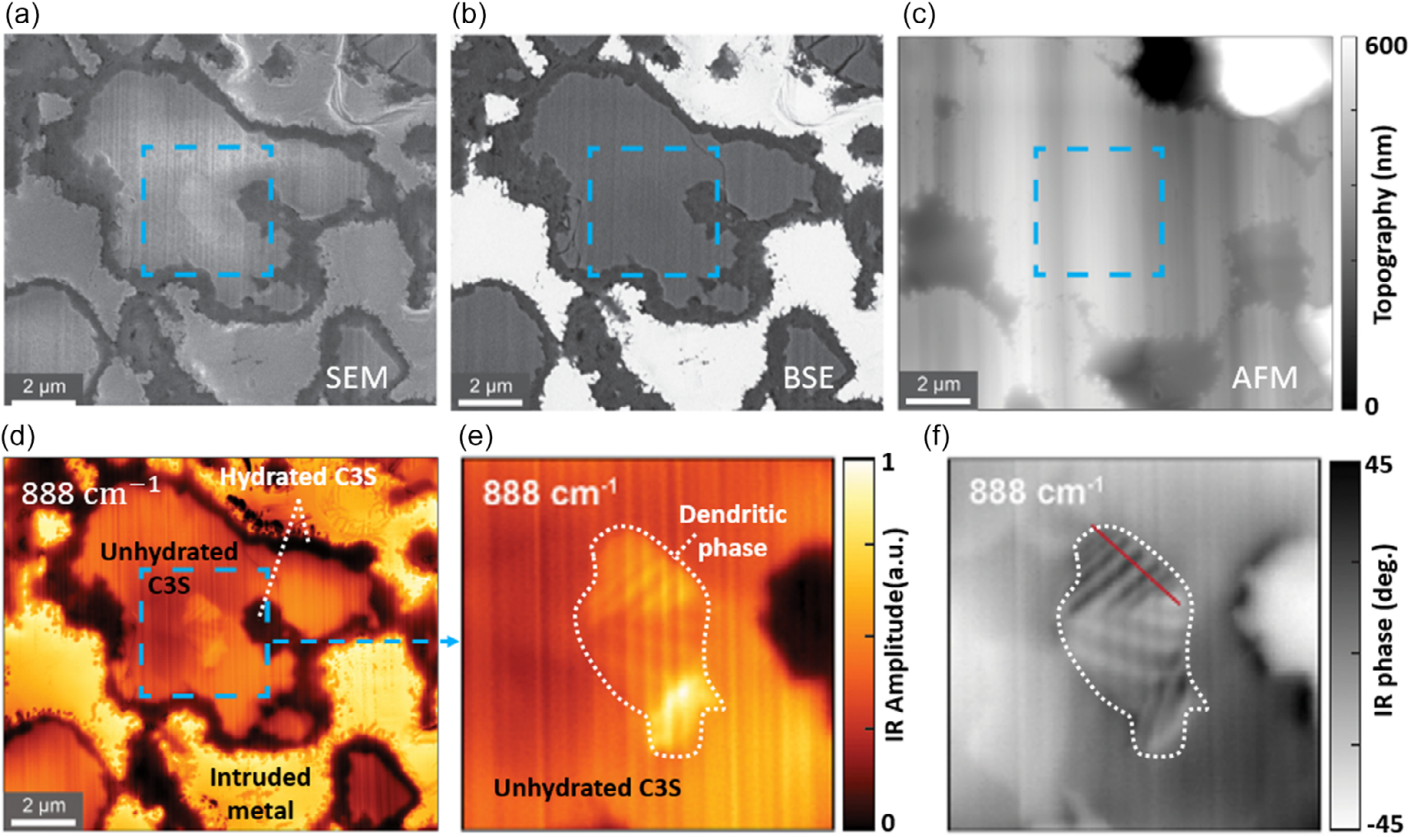
Characterizations of C3S cement sample. a,b) SEM images in a) SE and b) BSE modes; c) AFM topography and d) s‐SNOM amplitude image at 888 cm^−1^. Three main phases included: the intruded metal (high amplitude), unhydrated C3S (mediate amplitude), and hydrated C3S (low amplitude). e,f) The s‐SNOM amplitude and phase zoom‐in images of square area, uncovering a dendritic phase (marked by white dashed curves) within the unhydrated C3S grain. Reproduced with permission.^[^ ^119^ ^]^ Copyright 2021, Elsevier.

Various material phases are important and have distinct properties.^[^ ^116^, ^117^, ^118^ ^]^ Nanoscale material phases can be characterized by the scanning probe nano‐IR. A presentative example in **Figure** **7** showcases the nano‐IR's unique capability of visualizing nanoscale phases in tricalcium silicate (C3S) Portland cement^[^ ^119^ ^]^ over conventional approaches. While invisible in conventional nanoanalysis methods, including scanning electron microscopy (SEM) secondary electrons (Figure 7a), backscattered electrons (BSE, Figure 7b), and AFM (Figure 7c), the dendritic phase made of alternating ribbons of C3S and dicalcium silicate (C2S) was evidently imaged by the s‐SNOM (Figure 7d–f). At the C3S resonance at 888 cm^−1^, C2S ribbons exhibit a weaker IR absorption (lower phase in Figure 7f) than the nearby C3S, leading to fringes (with a period of ≈200 nm) in the s‐SNOM images (Figure 7e,f). Other phases, including the hydrated C3S, unhydrated C3S, and intruded metal can also be imaged due to their different conductivity and permittivity (Figure 7d).

### 3.7 Identification of Intracellular Structures

Intracellular structures, e.g., melanosomes, fibrillar components (FC), and nuclear acids, can be systematically studied by the scanning probe nano‐IR. Melanosomes—specialized organelles to produce melanin—were investigated on the sliced sheep air (**Figure** **8**a–f) without typical harsh chemical treatments in conventional methods.^[^ ^120^ ^]^ The intracellular melanosomes reveal evident characteristic resonances at 1422, 1467, 1540, and 1575 cm^−1^ for eumelanin (Figure 8a,b), 1124, 1172, and 1213 cm^−1^ for pheomelanin (Figure 8c,d), and the mixture of eumelanin and pheomelanin (Figure 8e,f), at different intracellular locations (Figure 8a,c,e).^[^ ^121^ ^]^

Figure 8Identification of intracellular structure. a,c,e) IR broadband images of melanosomes in the cortex of black sheep hair. b,d,f) The nano‐FTIR absorption spectra were measured at marked positions in a,c,e) and it reveals the melanosomes consist of pheomelanin, eumelanin, and the mixture of pheomelanin and eumelanin. Insets are the chemical formula of eumelanin and pheomelanin. Reproduced with permission.^[^ ^121^ ^]^ Copyright 2018, The Royal Society of Chemistry. g) s‐SNOM phase image of a single myeloma cell at the frequencies of amide I (1667 cm^−1^). Yellow arrows indicate nuclear membrane (NM) and nucleolus (Nuc). h) Zoom‐in phase image of dashed squares in g). A horseshoe‐shaped pattern at 1667 cm^−1^ marked by two yellow arrows indicating FC and DFC, respectively. i) Line profile of phase variations are extracted at the position shown in h); blue and yellow curves refer to 1667 and 1020 cm^−1^, respectively. j) s‐SNOM phase image of a single myeloma cell at the frequencies of C—O bonds (1020 cm^−1^). k) Zoom‐in phase image of dashed squares in j). Two cyan arrows point the regions with high absorption of C—O bonds, indicating high nucleic acid density. l) Line profile of phase variations are extracted at the position shown in k). Reproduced with permission.^[^ ^70^ ^]^ Copyright 2023, Springer Nature.

**Figure:**
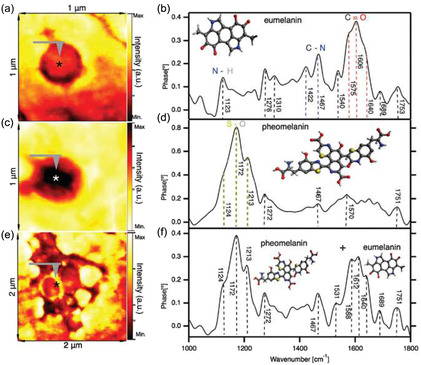

**Figure:**
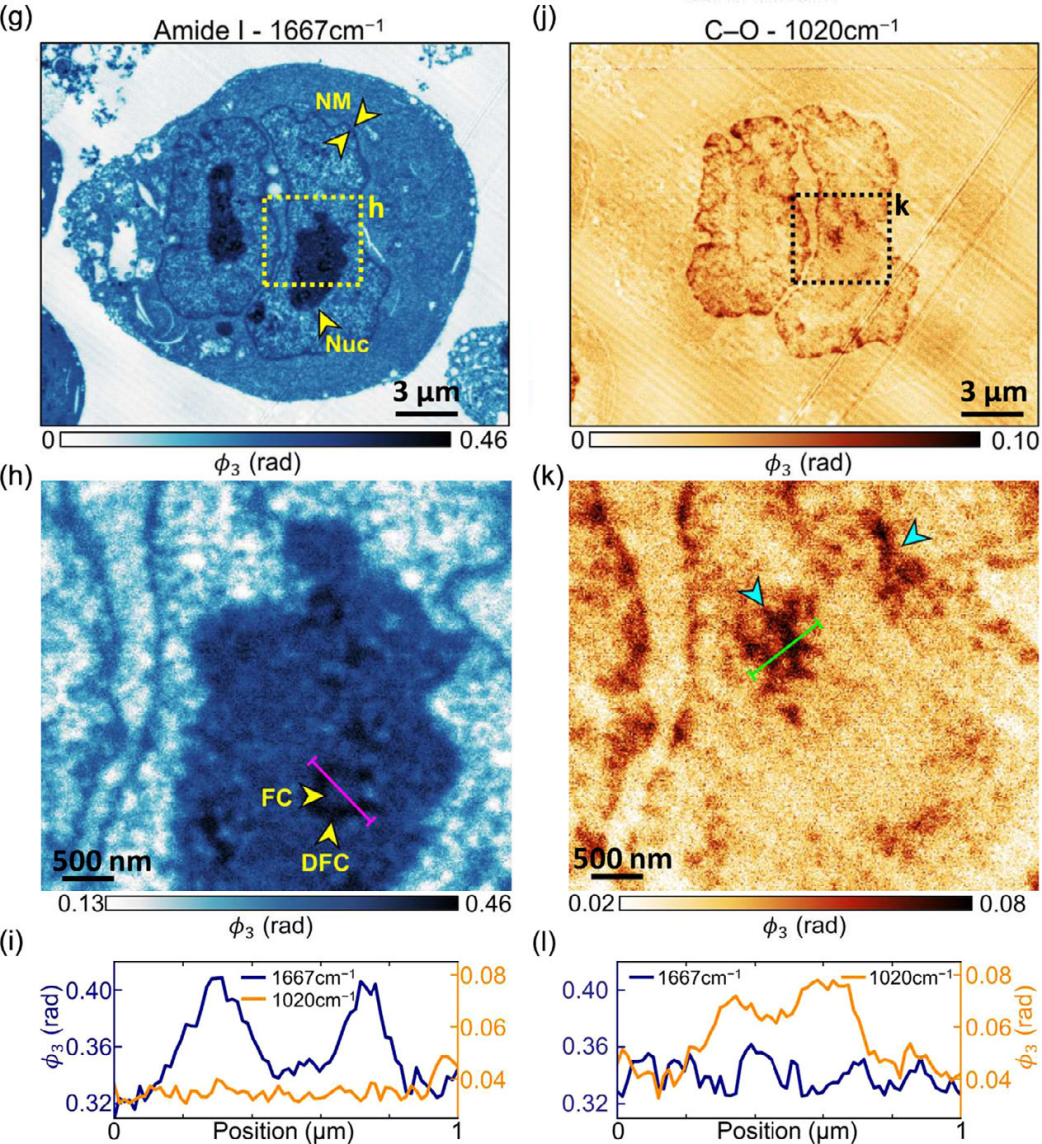

In addition to melanosomes, the intracellular heterogeneity of FC and nuclear acids can be imaged at their characteristic amide I (Figure 8g–i) and C‐O (Figure 8j–l) resonances, respectively. At FCs’ amide I resonance (Figure 8h), horseshoe‐shaped dense fibrillar components (DFC) exhibit a higher IR absorption (higher s‐SNOM phase *ϕ*_3_) than FC in the center. Membrane lipids and nuclear acids are rich in the C—O bond. At its resonance (1020 cm^−1^ in Figure 8j,k), the membrane lipids can be imaged (Figure 8j), and high‐density nuclear acids nanoregions can be identified in the s‐SNOM phase image (cyan arrows in Figure 8k) with a higher absorption (higher s‐SNOM phase *ϕ*_3_).^[^ ^70^ ^]^

**Figure 9:**
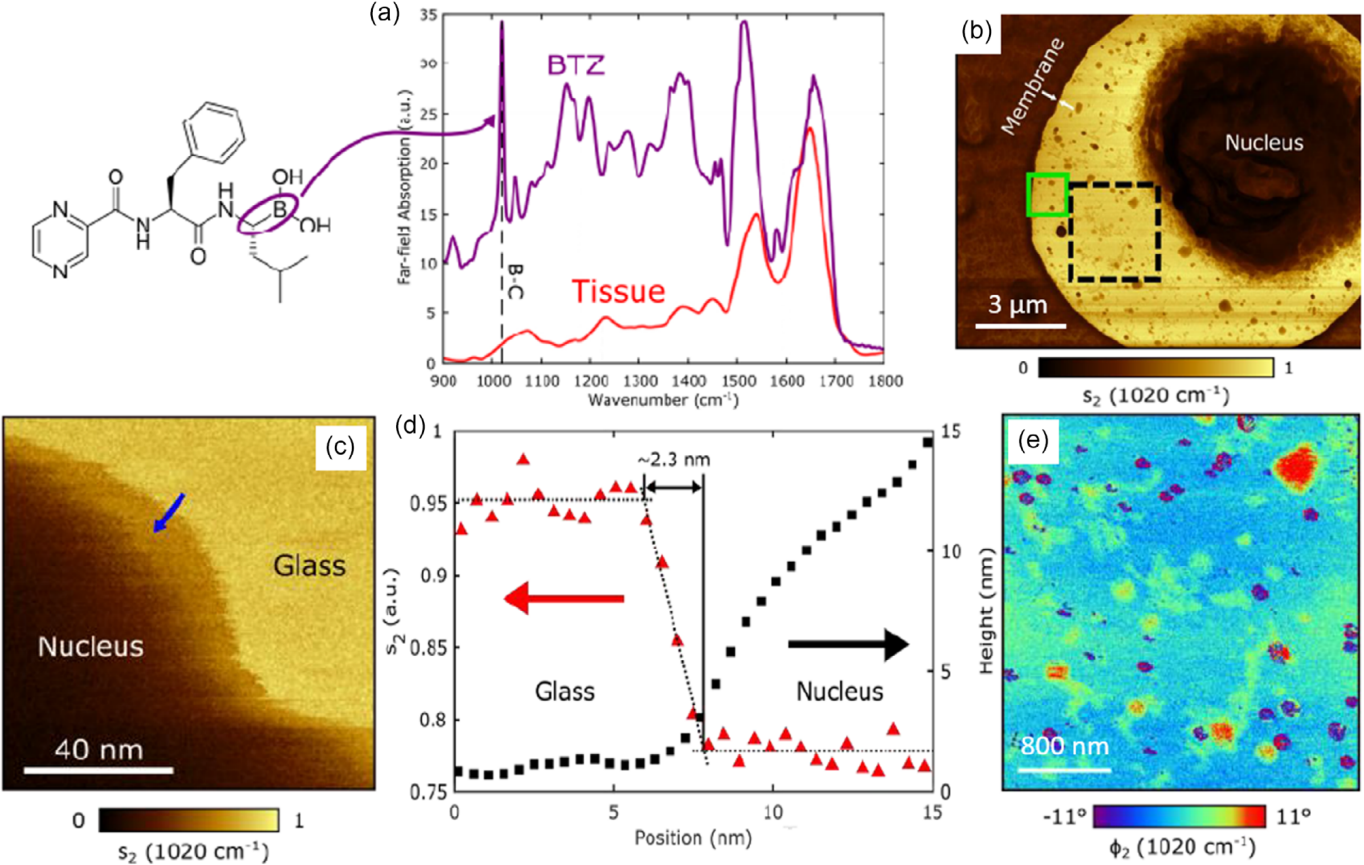
Identification of intracellular drug binding sites. a) The far‐field absorption spectra of BTZ and typical human tissue. b) The s‐SNOM amplitude (*S*_2_) image of a section of a human myeloma cell at 1020 cm^−1^. c) The zoom‐in view of a s‐SNOM amplitude (*S*_2_) image (1020 cm^−1^) at the boundary of cell nucleus. The low IR amplitude regions correspond to the nucleus of cell. The high IR amplitude regions refer to the glass substrate due to relatively larger reflection compared to nucleus. d) The line profiles of near‐field amplitude *S*_2_ (red triangles) and AFM topographical height (black squares) were extracted along blue arrow in (c). Glass regions have lower AFM heights but higher IR amplitude (*S*_2_) than nucleus regions. e) The phase image *ϕ*_2_ (1020 cm^−1^) at the dashed black square in (b). The areas with high phase contrast indicate intracellular BTZ drug binding sites. Reproduced with permission.^[^ ^122^ ^]^ Copyright 2020, arXiv.

Notably, in the s‐SNOM experiments, while simultaneously produced by the scanning probe, nano‐IR images may have a better resolution than the AFM topography because the former is based on the “lightening rod effect” confined at a length scale smaller than the tip radius that determines the resolution of the latter. This resolution difference was demonstrated in a recent study^[^ ^122^ ^]^ on bortezomib (BTZ) in human myeloma cells (**Figure** **9**). At the BTZ's characteristic B–C resonance (the 1020 cm^−1^ resonance in Figure 9a), the s‐SNOM line profile (red triangles in Figure 9d) reveals a resolution of ≈2.3 nm, surpassing the ≈20 nm resolution of the AFM data (black squares, Figure 9d). This ultrahigh nano‐IR resolution thus allows the imaging of intracellular BTZ distribution at the nanoscale (Figure 9e).

### 3.8 Protein Nanoscale Secondary Structures

**Figure 10:**
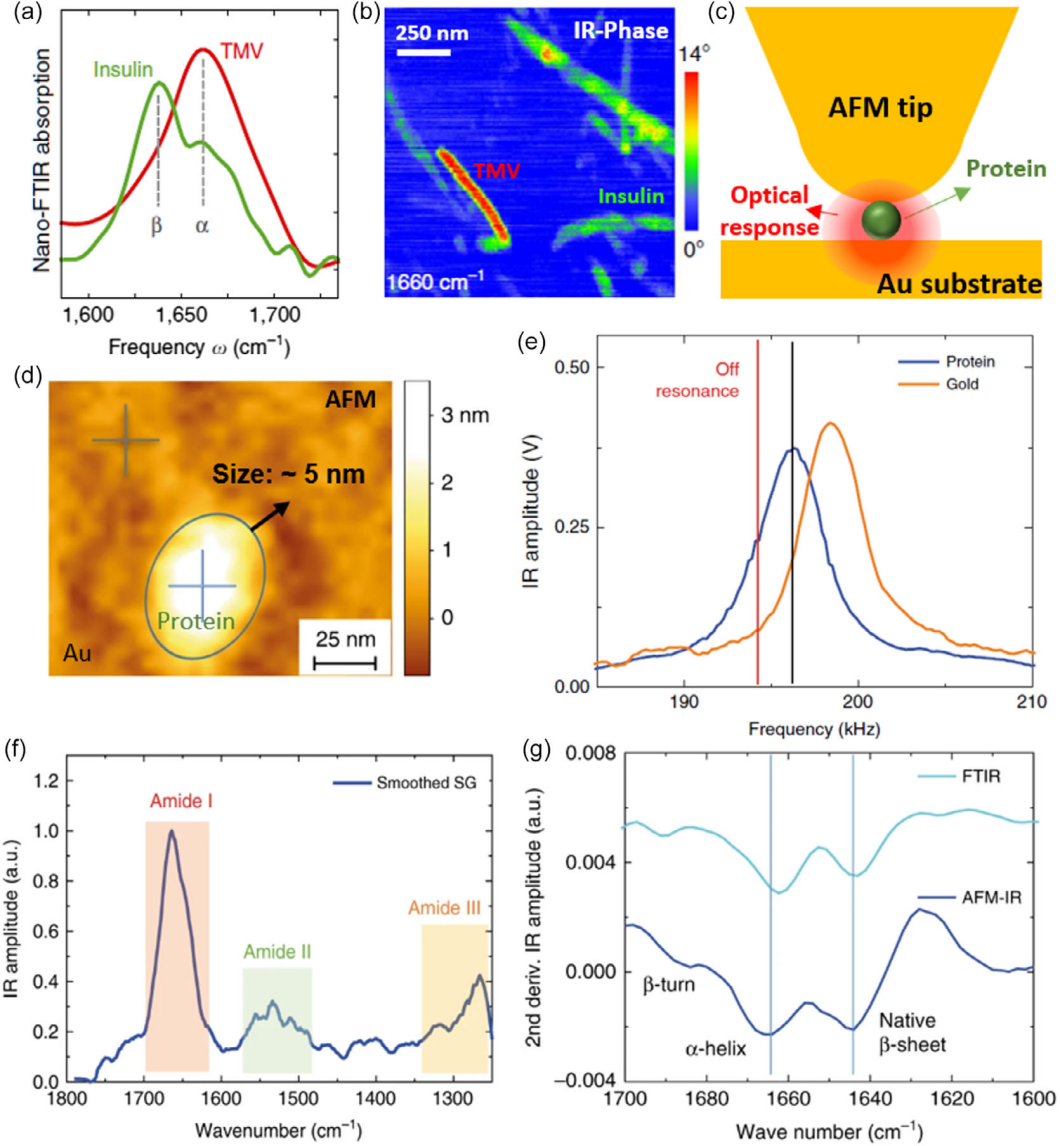
Identification of protein structures using nano IR imaging and spectroscopy. a) Nano‐FTIR absorption spectra for insulin (green) and TMV (red) aggregates in the amide I band. b) Near‐field phase images of a mixture of Insulin and TMV at 1660 cm^−1^ (characteristic frequency of α‐helix), where TMV has stronger contrast than insulin. Reproduced with permission.^[^ ^76^ ^]^ Copyright 2013, Springer Nature. c) AFM‐IR tip on a protein molecule (green circle). The volume of optical response (red region) beneath AFM tip. d) AFM image of a single thyroglobulin protein (blue circle) on gold substrate. The height of the protein molecule indicating the size ≈5 nm, which is highlighted by a black arrow. AFM‐IR spectra of protein and gold substrate are measured at the locations of “cross” markers. Lateral dimensions of protein are overestimated due to sample smaller than tip radius. e) IR amplitude (cantilever deflection) as a function of laser pulse frequencies on a protein particle (blue) and gold substrate (orange). The ratio between IR amplitude on protein and on the gold was improved from ≈1.9 (at black vertical line) to ≈2.7 (at red vertical line). f) AFM‐IR spectra of a single thyroglobulin protein measured and referenced at the “cross” marked positions in (d). This AFM‐IR spectrum identifies the amide I, II, and III bands of thyroglobulin. The spectrum was smoothed by Savitzky–Golay (SG) method. g) Second derivative of AFM‐IR spectrum within amide I band, shown detailed secondary structures of a single‐molecule thyroglobulin (navy blue curve). It agrees well with FTIR spectrum of bulk thyroglobulin samples (light blue curve). Reproduced with permission.^[^ ^72^ ^]^ Copyright 2020, Springer Nature.

Secondary structures affect protein's biological functions, stability, folding, and interactions with other molecules that are vital for diverse biological processes. They exhibit spectral resonances inside the mid‐IR amide I band.^[^ ^73^, ^74^, ^75^, ^76^, ^78^, ^123^, ^124^, ^125^ ^]^ Therefore, various proteins, including ferritin, viruses, bacteria, peptides nanosheets, amyloid fibrils, tobacco mosaic virus (TMV), proteins in lipids, proteins in purple membranes, and their characteristic secondary structures, can be studied in the nanoscale using the scanning probe nano‐IR imaging and spectroscopy. For example, insulin, predominantly composed of β‐sheet secondary structures, and TMV, mainly comprised of α‐helix secondary structures, were identified in nanoscale at characteristic frequencies of 1634 cm^−1^ (β‐sheet) and 1660 cm^−1^ (α‐helix, **Figure** **10**a,b).^[^ ^76^ ^]^

In addition, the nano‐IR investigation of protein secondary structures can reach very high precision by delicately controlling the illumination–tip interactions. For example, high nano‐IR sensitivity can be achieved by detuning the laser repetition to eliminate the background signal from the Au substrate.^[^ ^72^ ^]^ Typically, the scanning probe nano‐IR probes the optical response from a certain volume (Figure 10c), similar to the tip apex (length ≈20 nm). Therefore, for small specimens, e.g., the single‐molecule thyroglobulin of ≈5 nm, the AFM‐IR signal contains a considerable amount of background from the substrate (in this case, >50% from the Au substrate) at the resonant laser repetition (black line in Figure 10e). The signal from the protein nanoparticle can be purified by detuning the laser repetition (to the red line in Figure 10e) to obtain a precise spectrum of the secondary structures (Figure 10f). Furthermore, detailed secondary structure information (β‐turn, α‐helix, native β‐sheet, etc., in Figure 10g) can be extracted from the second derivative of the precise AFM‐IR spectrum.

### 3.9 Direct and Label‐Free Study of Cell Behaviors for Disease Diagnosis

**Figure 11:**
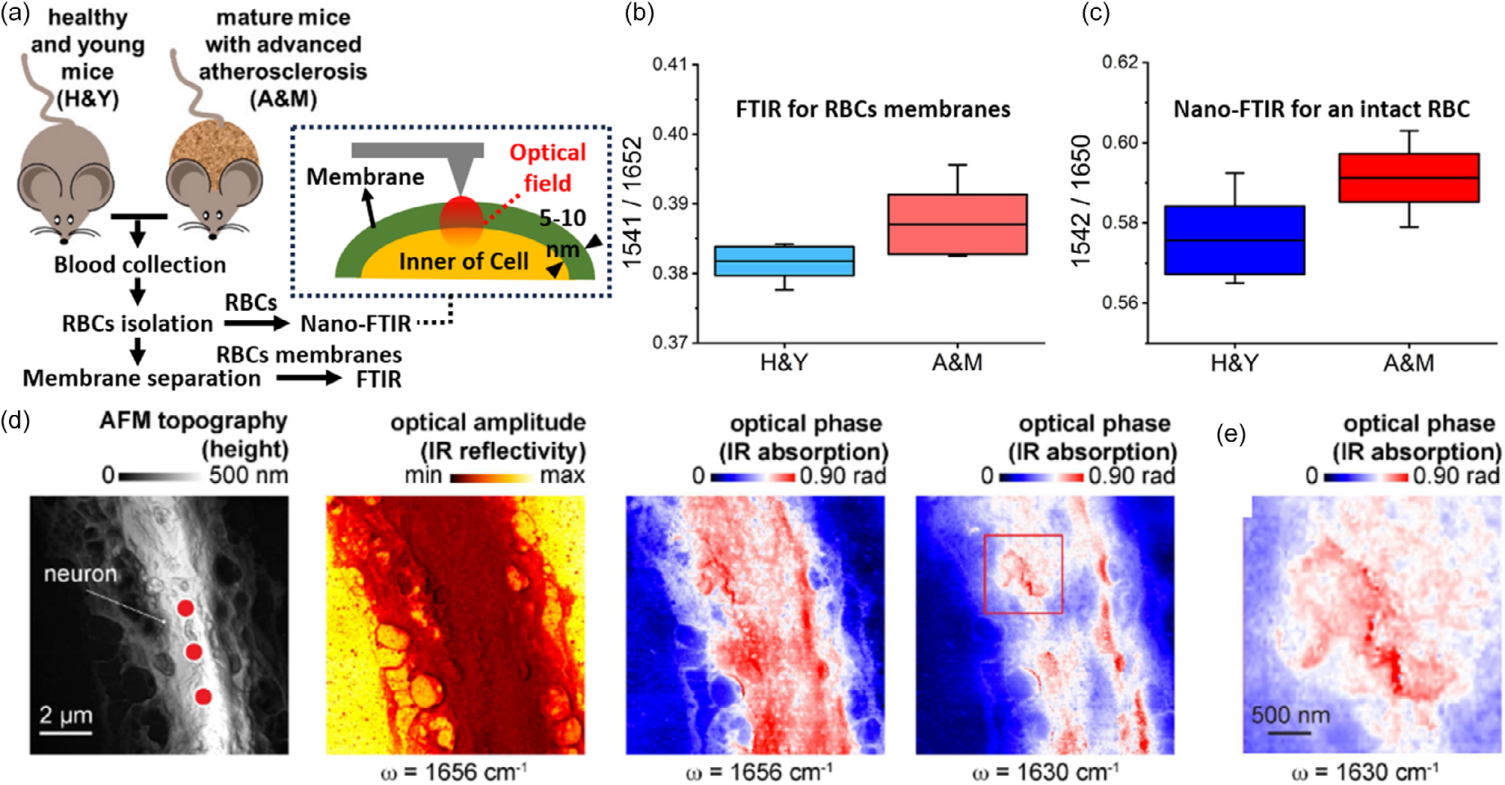
Identification of intact cells. a) The workflow for spectroscopic characterization of RBCs membranes. Inset: Schematic of nano‐FTIR on an RBC with optical field (red circle) beneath AFM tip mainly confined on membrane (membrane thickness 5–10 nm). Peak height ratios of amide II to amide I bands extracted from b) ATR‐FTIR on RBCs membranes and c) nano‐FTIR on intact cells, respectively. H&Y refers to RBCs of healthy and young mice. A&M refers to RBCs of mature mice with atherosclerosis. Reproduced with permission.^[^ ^126^ ^]^ Copyright 2019, American Chemical Society. d) AFM, s‐SNOM optical amplitude, and phase images of a single primary neuron at 1656 cm^−1^ (α‐helix) and 1630 cm^−1^ (β‐sheet). e) The zoom‐in optical phase image of red square in d), which shows the regions of aggregation of β‐sheet structures, indicating amyloid patches on neuronal surface. Reproduced with permission.^[^ ^71^ ^]^ Copyright 2021, MDPI.

Cell membranes—selective barriers between the cell interior and external environments—are crucial for cell functionality. Membranes’ structure change, as revealed in their mid‐IR fingerprints, may be used for disease diagnosis. Notably, cell membranes can be directly studied using the scanning probe nano‐IR without the complicated and/or destructive membrane isolation processes in conventional methods. This advance originates from the surface confinement nature of the scanning probe nano‐IR, where only the sample's top surface is examined (**Figure** **11**a).^[^ ^126^ ^]^ A representative example in Figure 11a–c showcases the diagnosis of atherosclerosis disease of mature mice (A&M) from healthy young mice (H&Y) by performing the scanning probe nano‐IR study of the red blood cells (RBCs) membranes (Figure 11a). The A&M exhibits a higher intensity ratio of amide II/amide I than the H&Y (Figure 11b). While it is convenient to diagnose membranes using the scanning probe nano‐IR, conventional FTIR spectroscopy has to rely on isolating cell membranes from the RBC because otherwise the mid‐IR signal from the hemoglobin‐dominated inner cell will mix with the genuine amide II and amide I responses from the membrane.

The scanning probe nano‐IR can also be used to image the aggregation of amyloid‐β protein to help diagnose Alzheimer's disease. Note that in immunofluorescence and immunoelectron microscopy, immunolabeling is mandatory yet potentially deteriorates the original specimen. Without such a procedure, the s‐SNOM phase image at the β‐sheet resonance (1630 cm^−1^ in Figure 11d) directly visualizes the aggregation of β‐sheets on the surface of a neuron cell. These results demonstrate a direct and label‐free nanoimaging of important protein structures in cells with complicated sample preparations and potential deterioration in conventional immunolabeling methods.^[^ ^71^ ^]^

### 3.10 Scanning Probe IR Nanolithography

**Figure 12:**
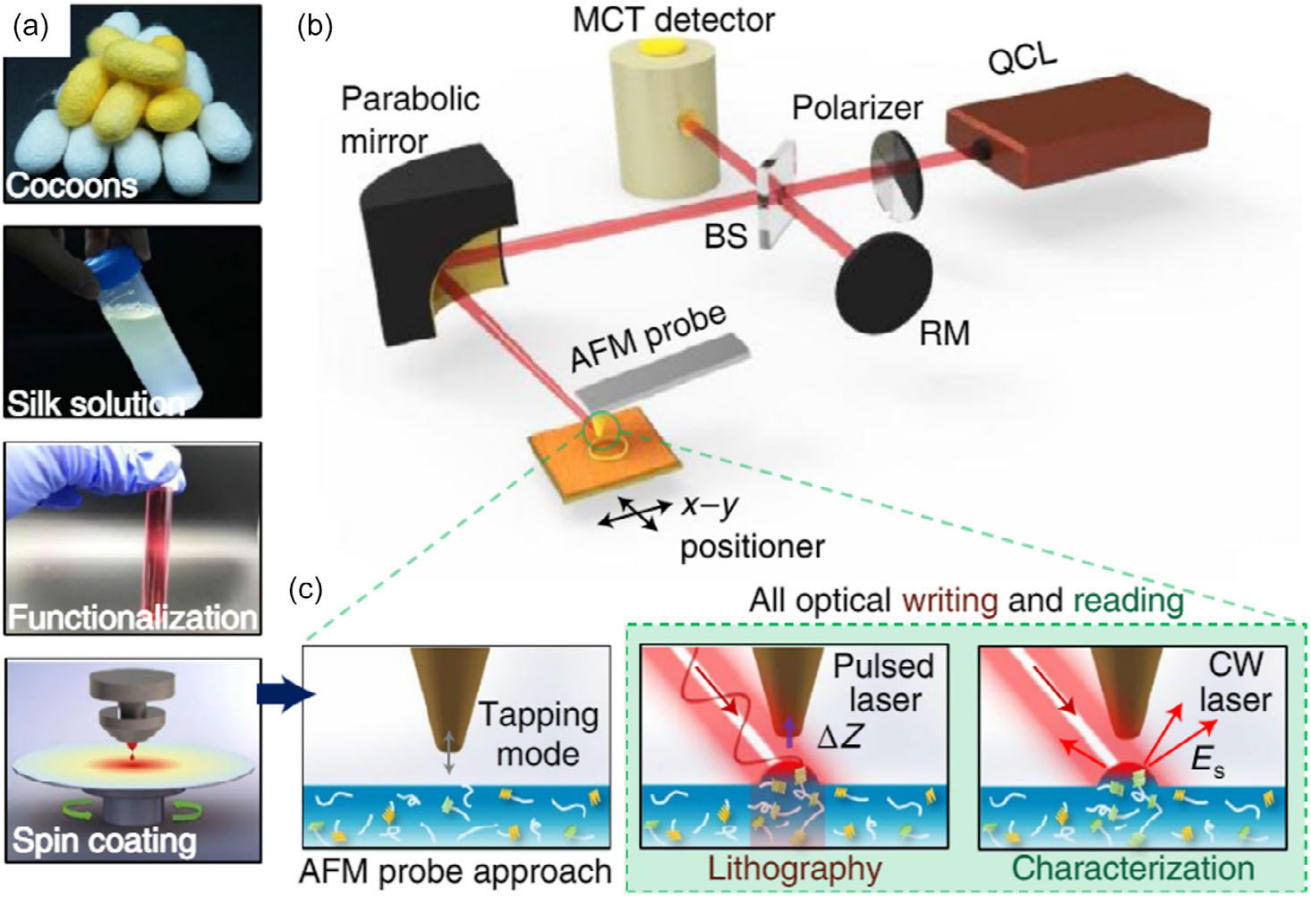
Scanning probe IR nanolithography on silk protein. a) Sample preparations. b) The schematic of s‐SNOM. c) Working flows of nanopatterning and characterization of silk film. Pulsed laser with the frequency of amide I band was used for nanolithography on silk film. CW laser was used for characterization nanopatterns at β‐sheet characteristic frequency (1631 cm^−1^). Reproduced with permission.^[^ ^127^ ^]^ Copyright 2020, Springer Nature.

In addition to nano‐optical characterizations, the scanning probe nano‐IR can be used for nanomanipulation and nanolithography. A representative example in **Figure** **12** showcases the scanning probe nano‐IR lithography on silk.^[^ ^127^ ^]^ The pulsed IR illumination at the tip locally heats the underneath silk protein, transforming its secondary structure from the amorphous state to β‐sheets. These heat‐induced β‐sheets aggregate toward the heat source (tip) and form an elevated “hill” (Figure 12c). When using stronger illumination, these β‐sheets nano “hills” thermally degrade and return to the amorphous silk with flat topography—the “erasing” effect. Therefore, the scanning probe nano‐IR can perform nanolithography on the silk protein in a rewritable fashion by controlling the IR illumination.^[^ ^127^, ^128^ ^]^

### 3.11 In Vivo Nano‐IR Characterization in Aqueous Environments

**Figure 13:**
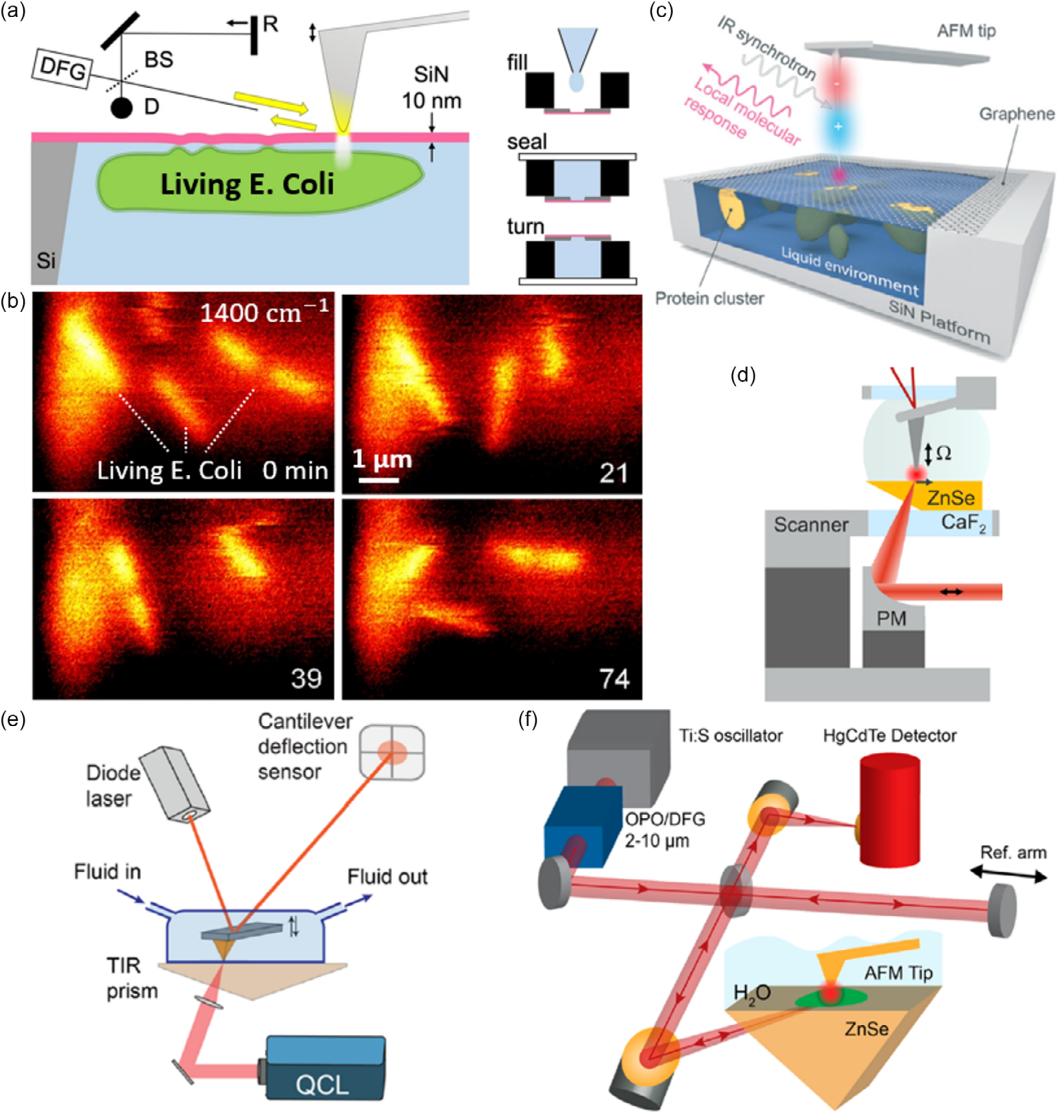
Measurements in liquid environment. a) Setup of SiN‐membrane‐based s‐SNOM. DFG, difference‐frequency generation; D, detector; BS, beam splitter; R, reference mirror. Right panels refer to the sample preparation process. b) Amplitude images of s‐SNOM at 1400 cm^−1^ for living *E. coli* cells under a SiN membrane, at times in minutes indicated. Image size 7.5 × 5 um^2^. Reproduced with permission.^[^ ^129^ ^]^ Copyright 2021, Springer Nature. c) Scheme of the s‐SNOM of protein cluster attached to a graphene monolayer in water. Reproduced with permission.^[^ ^131^ ^]^ Copyright 2019, The Royal Society of Chemistry. d,f) Two setups of TIR s‐SNOM. Reproduced with permission.^[^ ^134^, ^135^ ^]^ Copyright 2020, arXiv; Copyright 2020, American Chemical Society. e) Schematic of the LiPFIR microscope. Reproduced with permission.^[^ ^133^ ^]^ Copyright 2021, American Chemical Society.

Fundamental biomedical processes usually rely on molecules’ arrangement and dynamic interactions in aqueous environments. However, IR illumination and detection through the aqueous environments are strongly attenuated, and water's absorption resonances may overlap with important bio‐fingerprints in the mid‐IR, thereby making in vivo characterization challenging using the conventional scanning probe nano‐IR setup. This challenge can be mitigated by incorporating a thin membrane or bottom illumination (**Figure** **13**). Using a thin and IR transparent membrane (SiN and graphene in Figure 13a,c, respectively), adequate IR illumination and scattering are allowed for in vivo imaging of the living motion of the *Escherichia coli* (*E. coli*, Figure 13b)^[^ ^129^ ^]^ and protein dynamics (Figure 13c).^[^ ^130^, ^131^, ^132^ ^]^ Alternatively, in order to obtain the concurrent topographic and mechanical responses of the sample in aqueous environments, an IR‐transparent ZnSe lens can be placed under the sample to enable bottom illumination during the scanning probe nano‐IR experiments. PFIR^[^ ^133^ ^]^ and s‐SNOM^[^ ^134^, ^135^ ^]^ could be performed under this setup (Figure 13d–f) in aqueous environments on biodevices, polymers, and polaritonic media.

## 4 Summary and Prospects

By enhancing the local tip–sample interactions, various scanning probe nano‐IR techniques enable access to material optical and mechanical responses with a spatial resolution down to 10 nm. This advance has been utilized to probe nanoscale material composition, heterogeneity, orientation, fine structure, and phase transitions in a variety of specimens, including polymers, natural materials, organics in soil and petroleum, intracellular structures, and protein secondary structures. The IR illumination of the scanning probe can also be delicately controlled for erasable nano‐IR lithography. In addition, by incorporating the IR transparent membrane and bottom illumination, scanning probe nano‐IR in aqueous environments became possible for in vivo characterizations of living biomedical systems.

The progress highlighted in Sections 2 and 3 showcases the scanning probe nano‐IR as a superb tool for characterizing bio and natural specimens at the nanoscale. Building on these advancements, we anticipate future explorations in the following example areas: 1) Polarization‐unlimited scanning probe nano‐IR. The current scanning probe nano‐IR typically probes the p‐polarized optical responses due to the vertically placed AFM tip (Figure 1b–d). Yet, polarization and directionality are important parameters in optics and light–matter interactions. Future works may be directed toward the scanning probe nano‐IR characterization with tips or optical enhancements along other directions to probe nano‐optical responses or nanoscale light–matter interactions with various polarizations. The polarization‐unlimited scanning probe nano‐IR can, therefore, become a comprehensive nanoscale analysis tool for a wide range of optical responses at advanced length scales; 2) Better spatial resolution toward the atomic scales. Although it was already a milestone that the scanning probe nano‐IR broke the diffraction limit to offer optical characterizations at ≈10 nm, a fruitful of optics phenomena and material optical responses remain unexplored at even smaller length scales approaching the atom sizes. While sharper scanning probes, e.g., carbon nanotube, can yield a better resolution, these probes’ miniature scattering cross sections may not offer adequate scattering signals for practical detection. One potential strategy may involve electromagnetics computation or machine learning to model the atomic scale tip–sample responses in the scanning probe nano‐IR experiments. In a reversed fashion, these computational algorithms may be utilized to improve the spatial resolution of scanning probe nano‐IR by extracting optical and mechanical signals smaller than the experimental input (tip radius, ≈10 nm) to approach the sub‐nanometer or atomic scale optical responses; 3) Nano‐IR characterizations of genuine biological functionalities and living specimens. Genuine properties and functionalities of biological specimens are difficult to stay unaffected once isolated from intact biological systems. Therefore, preservable sample processes, such as formalin‐fixing,^[^ ^122^ ^]^ paraffin‐embedding, and other advanced techniques, may be explored in scanning probe nano‐IR characterizations to probe the genuine biological functionalities in practical specimens. Based on the pioneering developments of IR‐transparent isolation membrane on top^[^ ^129^, ^130^, ^131^ ^]^ or bottom illumination through properly designed prisms,^[^ ^133^, ^134^, ^135^ ^]^ the scanning probe nano‐IR may be utilized to characterize a wide range of living systems in their original aqueous environments, including pH solutions, ionic solutions, high‐viscosity liquids, and even microfluids; and 4) Nanoscale dynamics in biomaterials and devices. In addition to static properties when specimens are settled down at specific periods, dynamic responses dominate most of the time, especially for living biomaterials and devices. Specifically, microscale motions of living cells occur in seconds.^[^ ^136^ ^]^ Protein dynamics are even faster at various time scales. For example, the protein structure changes due to the chemical bonds variations and side‐chain motions occur in femtoseconds (10^−15^ s) to picoseconds (10^−12^ s).^[^ ^137^, ^138^ ^]^ The dynamics of active site residues in enzymes are in picoseconds to nanoseconds (10^−9^ s),^[^ ^139^ ^]^ whereas protein domains typically move in microseconds (10^−6^ s) to milliseconds (10^−3^ s).^[^ ^137^, ^138^ ^]^ It is, therefore, crucial to investigate the nanoscale dynamics of biomaterials and devices. However, a standard scanning probe nano‐IR scan takes at least several minutes and cannot capture most dynamic responses. Possible solutions may be to exploit fast AFM^[^ ^69^ ^]^ and improve the signal‐to‐noise ratio (thus less signal integration time) in scanning probe nano‐IR to image the microscale motions of living cells. In addition, ultrafast IR pulses may be utilized to illuminate the scanning probe for pump‐probe studies of photoinduced dynamics in protein and other biomaterials and devices.

## Conflict of Interest

The authors declare no conflict of interest.

## Author Contributions

**Jialiang Shen**: Writing—original draft (lead). **Byung‐Il Noh**: Writing—original draft (supporting). **Pengyu Chen**: Writing—review & editing (supporting). **Siyuan Dai**: Conceptualization (lead); Funding acquisition (lead); Supervision (lead); Writing—original draft (supporting); Writing—review & editing (lead).
